# Laser surgery reveals the biomechanical and chemical signaling functions of aphid siphunculi (cornicles)

**DOI:** 10.1371/journal.pone.0204984

**Published:** 2018-10-08

**Authors:** Serine Alfaress, Craig R. Brodersen, El-Desouky Ammar, Michael E. Rogers, Nabil Killiny

**Affiliations:** 1 Citrus Research and Education Center, IFAS, University of Florida, Lake Alfred, FL, United States of America; 2 School of Forestry & Environmental studies, Yale University, 195 Prospect, New Haven, CT, United States of America; 3 United States Department of Agriculture, Agricultural Research Service, US Horticultural Research Laboratory, Fort Pierce, FL, United States of America; University of Vigo, SPAIN

## Abstract

Aphids are an attractive food source to many predators and parasitoids because of their small size, soft bodies and slow movement. To combat predation, aphids evolved both behavioral and chemical defensive mechanisms that are operated via siphunculi (cornicles), differently developed structures that more or less extend from their abdomen. Although both direct and indirect linkages between siphunculi and their defensive mechanisms have been explored, their ultimate effects on aphid fitness are still broadly debated. To explicitly test the influence of siphunculi on brown citrus aphid, *Aphis* (*Toxoptera) citricida* (Kirkaldy), fitness, we razor-cut and laser-sealed the siphunculi. Siphunculi removal resulted in two distinct behavior modifications, (false aggregation and increased drop-off rates) that led to decreased survival and the loss of the ability to right themselves from an inverted position. These results together indicate that siphunculi play an important role in survival, and removal of these organs will have negative effect on aphid fitness. Furthermore, results suggested that released alarm pheromone may play an important role in communication among aphid clone-mate, and omitting it results in miscommunication and competition among clonemates. These findings will help in better understanding the aphid biology.

## Introduction

As aphids (Hemiptera, Aphididae) aggregate in high-density colonies on the stems and leaves of host plants, they are highly vulnerable to predation [1, 2]. To defend themselves, aphids have evolved physical, behavioral and chemical defensive mechanisms [2, 3] that appear to be linked to both the preservation of the individual and the colony, which likely stems from their parthenogenetic life history [4, 5]. A unique morphological structure of aphids that appears to play a major role in these defense mechanisms are the siphunculi (or siphunculi), two unique tube-like structures found only in aphids [6], and located dorsally/dorso-laterally on the posterior part of the abdomen [1, 6]. Siphunculi secrete two types of defensive compounds–a sticky droplet that binds the appendages and mouthparts of predators [7, 8] and an alarm pheromone, trans-β-farnesene ((*E*)-β-farnesene) used for chemical signaling [9]. Siphunculi have received significant attention from researchers, and rightly so: aphids are a significant agricultural pest and have also become model organisms for studying the ecology of predator-prey relationships [10]. While many studies hypothesize the direct or indirect fitness benefits provided by siphunculi, no definitive research shows the direct impact of the presence or absence of siphunculi on aphid survival and fitness. Investigating the effect of siphunculi removal will help in exploring their impact on aphid biology.

Recent research suggests that siphunculi evolved to provide direct fitness benefits to the individual aphid [11], primarily within the context of siphunculi droplet production deterring the advance of natural enemies [5, 12]. Aphids mostly feed on phloem sap [13], and their diet does not contain lipids [5, 12], which are essential for development and reproduction [5]. Secreting even a single cornicle droplet, therefore, may deplete limiting resources and negatively impact aphid fitness, especially for immature aphids [5]. Indeed, several studies suggest that cornicle droplet production may be less widespread than commonly assumed and is only deployed in rare circumstances [2, 14–16]. For example, only a small fraction of *Aphis fabae* (Scopoli) individuals out of several thousand were observed immobilizing hymenoptera parasitoids by daubing them with cornicle droplets [7], suggesting that this defensive behavior is not often used.

Other studies have refuted the role of cornicle droplets in direct fitness gains and suggest instead that an individual aphid often deploys droplets as part of an altruistic behavior [17]. For example, individual aphids have been reported to warn clone members of impending predation through alarm pheromones embedded in droplets, thus providing indirect fitness benefits [11, 12]. Alarm pheromones provide inclusive fitness through chemical signaling both locally [4, 11, 17–21] and beyond their feeding range by scent-marking predators with pheromone-laced droplets that are then carried by the predator as it forages for additional aphids [12, 17, 22]. In the case of the highly volatile alarm pheromone trans-β-farnesene, rapid diffusion through the air from the droplet [2, 16] can provide functional signaling for about 20 minutes after release [16, 22] with an effective range of approximately 3 cm [2, 16, 17].

In addition to secreting sticky cornicle droplets and alarm pheromone production, behaviors such as dropping off the plant and fleeing are also recognized escape behaviors in aphids [2]. For aphid living in groups, however, different studies suggested that drop-off behavior may incur a significant fitness cost for escaping aphids rather than fitness benefits because escaping aphids must expend additional energy to relocate to new feeding locations [23, 24]. This fitness cost can become substantial given the host specificity in many aphid species [24]. Ultimately, aphids escape natural enemies using several strategies, all of which have fitness costs and there are tradeoffs from one to another.

The defensive function of siphunculi has been accepted broadly by many aphidologists [1, 2, 16, 25], and is still the most accepted hypothesis. Whether siphunculi evolved for individual protection (direct fitness) or to enhance clone-mates survivorship (indirect fitness) remains widely debated. It is believed that aphid ancestors did not have siphunculi [11], so several key questions to be addressed are 1) what are the effects of cornicle removal on aphid behavior and fitness; and 2) can their biological functions (emission of defensive droplets and/or alarm pheromone) be determined by blocking them partially or completely? The current study, using the brown or tropical citrus aphid, *Aphis* (*Toxoptera) citricida* (Kirkaldy) (Aphididae), the most efficient vector of *Citrus tristeza closterovirus* (CTV), addresses these questions using novel methods to remove the siphunculi and block their functions.

## Materials and methods

### Aphid colonies

Colonies of *A*. *citricida*, were reared in a growth room inside mesh cages (35 x 35 x 60 cm) on 30-cm high (12 months old) *Citrus macrophylla* orange seedlings with multiple new shoot growth. *C*. *macrophylla* plants were grown in a greenhouse approved by USDA-APHIS (United States Department of Agriculture-Animal and Plant Health Inspection Service). Both the growth room and greenhouse had similar controlled conditions; 25° C temperature, 65% humidity and 16L: 8D photoperiod.

### Cornicle removal and laser sealing

Only apterous (wingless) viviparous adult females were used in this study. First, aphids were examined morphologically under a stereomicroscope (Trinocular 2X-225X extreme wide field zoom, Amscope, Irvine, CA) to make sure that all aphids were healthy-looking and of similar size and age (adults). Three different treatments were involved in this study: control, in which siphunculi were left intact; razor treatment, in which both siphunculi were removed using a sharp razor blade; and laser treatment, in which both siphunculi were removed with a razor blade and then sealed with a laser beam focused on cornicle area for two seconds. To seal the open hole in the abdomen we used a DM7050 standard professional diode laser system module (130 J∙mm^2^ 808 nm pinpoint beam, Biotechnique Avance, Oxford, England). The laser beam (1.5 mm) was used with the wavelength of 600 to 900 nm and adjusted at 65 J∙mm^2^/sec (half power). After treatments, BCA were examined again under the stereomicroscope individually to make sure that siphunculi were removed correctly and laser-sealed with no damage to any other part of the aphid’s body. Treated aphids were then placed on citrus plants for 24 h to make sure that they survived and were feeding normally.

### Scanning electron-microscopy (SEM) of aphids

SEM was used to study the abdomen’s structure in the area of the cornicle for control, razor, and laser treated aphids. First, treated or control aphids were dehydrated in 70% then 100% ethanol (two changes) before air-drying. Each aphid was then carefully placed, dorsal side up, using fine-pointed forceps (Fontax No. 5; Electron Microscopy Sciences, Washington, PA) under a stereomicroscope, on black conductive double-sided adhesive discs (9–12 mm diameter) on aluminum stubs (SPI Supplies, West Chester, PA). Aphid specimens on these stubs were then coated with gold-palladium using a Hummer 6.2 Sputter Coater (Anatech USA, Union City, CA) for 50 sec. Coated aphids were examined using a scanning-transmission electron microscope (Hitachi S-4800, Hitachi, Pleasanton, CA), in the SEM mode at 5 or 10 kV at magnifications of 100X and 400,00X. All electron micrographs were saved on image management software (Quartz PCI version 8) connected to the electron microscope. Ten aphids per treatment were examined by SEM.

### High resolution X-ray Micro-computed Tomography Imaging (microCT)

To verify the effects of the surgical removal of siphunculi we scanned four aphids from each treatment with microCT imaging, a non-invasive imaging tool based on X-ray absorption. Scanning was performed at the Lawrence Berkeley National Laboratory Advanced Light Source, beamline 8.3.2 following the methods of Brodersen et al. [26]. Briefly, aphids were prepared as above for SEM imaging, but were not sputter coated. This sample preparation allowed for the visualization of internal structures in the cornicle area to confirm the complete blockage of the cornicle aperture in the laser-treated aphids. Individual aphids were loaded into a polyamide tube and mounted in the microCT system and imaged at a 3.25μm voxel resolution at 24 keV. The raw tomographic images were reconstructed with Octopus software (University of Ghent, Ghent, Kortrijk, Belgium) and visualized with Avizo software (VSG Inc., Burlington, MA).

### Gas chromatography–mass spectrometry (GC-MS)

#### Collecting alarm pheromone

Alarm pheromone (trans-β-farnesene) was collected from *A*. *citricida* as reported previously [27] in order to examine the difference in abundance of collected alarm pheromone among the three cornicle treatments mentioned above. The method was created to collect the alarm pheromone from living aphids, as the crushing method (classical method) may allow the body volatiles to be released while we are testing whether cutting and laser sealing the siphunculi prevents the release of alarm pheromone. In each treatment, replicated five times, aphids were exposed to the same following conditions; 36 mg (35 to 40 individuals) wingless adults were placed in glass vials adapted for solid phase micro extraction (SPME) and the fiber (1 cm Carb/DVB/PDMS, Supelco, Bellefonte, PA) was inserted inside the vial to collect the volatiles. Aphids were first exposed to cold microclimate conditions by positioning the vial with aphids over a beaker filled with ice (without touching) for 6 h. Immediately following the cold treatment, aphids were exposed to a hot microclimate conditions created by moving the vial, again without touching, over a hot water bath set at 40°C and for 12 h; after the temperature exposures, the fiber was removed from the vial and desorbed directly into the GC-MS for volatile analysis.

#### GC-MS analysis

To analyze released volatiles in samples and authentic standards we used a Clarus 680 GC-MS system (Perkin Elmer, Waltham, MA). The GC-MS was provided with a ZB-5 column (cross-linked 5% Phenyl-95% Dimethylpolysiloxane, 30 m × 0.25 mm × 0.25 μm film thickness) and hydrogen carrier gas with a flow rate 1 ml/min. The injector liner was 2 mm and samples were desorbed for 5 min, in splitless mode. The oven temperature program was as follows; initial temperature was programmed to start at 70°C for 1 min, then increased to 220°C at a rate of 10°C/min held for 1 min, and then the final temperature increased to 300° C at 10°C/min. The MS detector temperature was 180°C and the injector temperature was 250°C.

#### Peak identification

TurboMass software version 5.4.2 (Perkin Elmer, Waltham, MA) was used to analyze GC-MS chromatograms. NIST mass spectra library (National Institute of Standards and Technology, Gaithersburg, MA, USA); Wiley 9th edition (John Wiley and Sons, Inc., Hoboken, NJ) mass spectra database libraries were used to identify trans-β-farnesene. Both retention time and mass spectra of trans-β-farnesene were compared with those of the authentic reference standard to confirm identification.

### Biomechanical role of siphunculi experiment

To examine the biomechanical role of siphunculi, three treatments (control, removal of two siphunculi with razor, removal of one siphunculus with razor) were evaluated. Razor-treated aphids were left on the plant for 24 h before conducting the experiment. In each treatment, an aphid was flipped on its back with a paintbrush and the time required for each aphid to return to its normal upright position was recorded. Body parts that the aphid used to return to its normal position were also observed. Each treatment was replicated ten times.

### Fitness experiments

Aphids were placed on plants either individually (one aphid/ plant) or in groups (10 aphids /plant). In experiments of grouped aphids, each aphid was placed separately on a different leaf to avoid physical contact between aphids. In all experiments, the plant soil surface was covered with white filter paper to observe escape or drop-off behavior. Offspring were removed daily from all plants after counting to avoid crowding in colonies. Experiments were repeated three times and continued until adult(s) died.

#### Fitness of individual aphids (one aphid/plant)

This experiment was conducted to study the effects of cornicle removal on the fitness of individual aphids. Treatments included groups (control, razor, laser) described earlier. After treatment, each individual aphid was placed on one plant. Two parameters of fitness including mother-aphid survival and the number of offspring were recorded daily. Escape or drop-off behavior was also investigated. All treatments had 12 replicates.

#### Fitness of aphid groups (10 aphids/plant)

To study the effects of cornicle removal on indirect fitness, we performed an experiment similar to the individual fitness experiment but in this case, 10 aphids were confined to each plant with five replicate plants per treatment. Fitness parameters including number of offspring, mother survival, aggregation, and drop-off behaviors were all evaluated in this experiment. For mother survival, dropped-off aphids unable to return to the feeding area were counted as alive until they desiccated or died.

### Survival of mixed treatments (5 Control-5 Razor or Laser treated) aphids

To study the effects of aggregation behavior and chemical communication on aphid fitness, we conducted an experiment in which five control aphids were placed on one plant with five-cornicle-treated (razor or laser) ones. Each aphid was placed on a different leaf. Aggregation and drop-off behaviors were investigated. Mother survival was recorded daily which was followed by offspring removal. Number of offspring parameter was not considered because it was not possible to differentiate between the offspring of control and treated aphids.

### Statistical analysis

Data were analyzed using Minitab^®^ 16 software (State College, PA). Analysis of variance (ANOVA) followed by Tukey post hoc test were performed to compare number of offspring among control, razor, and laser treatments. Kaplan-Maier survival analysis was used to compare mother survival among the three above mentioned treatments.

## Results

### Examination of cornicle structure and function

#### Scanning electron microscopy (SEM)

**Fig 1:**
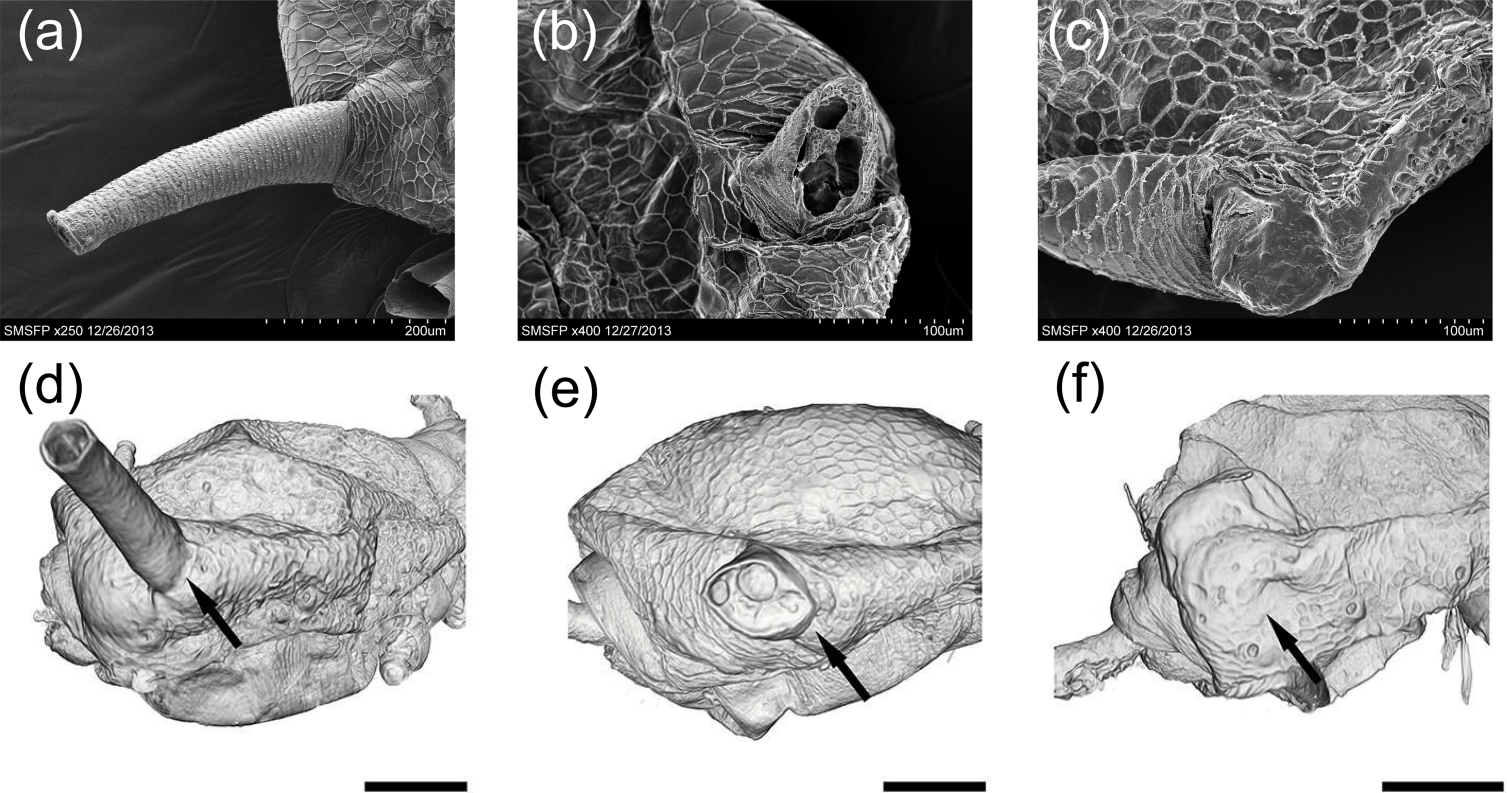
Scanning electron microscopy (SEM) and high resolution X-ray Micro-computed Tomography Imaging (microCT) of cornicle area before and after removal with razor and/or laser. SEM micrograph of a control aphid with intact siphunculi (only one cornicle is shown here) (a). SEM of a razor treated aphid with removed siphunculi and damaged open cornicle area (b). SEM of a laser treated (cut with razor then sealed with laser beam) showing no cornicle and fully sealed cornicle area (c). microCT image of a control aphid with intact siphunculi (d). microCT image of a razor treated aphid with removed siphunculi and damaged open cornicle area (e). microCT image of a laser treated (cut with razor then sealed with laser beam) showing no cornicle and fully sealed cornicle area (f).

The two siphunculi and surrounding areas of brown citrus aphid were examined using SEM for control, razor, and laser treated aphids. SEM images of control aphids showed that the length of an intact sephunculus is approximately 260 μm, while its width is about 55 μm at the tip and 100 μm at the base (Fig 1A**)**. In the razor treatment, both siphunculi were completely removed; while the basal areas of siphunculi remained open and cutting damage of razor was obvious **(**Fig 1B**)**. Siphunculi treated with laser were also completely removed, but the cut areas were fully closed indicating the efficacy of sealing with laser surgery **(**Fig 1C**)**.

#### microCT imaging

The microCT images of the control, razor, and laser treatment groups confirmed the SEM imaging data. Complete removal of the siphunculi using the razor and laser treatments was evident both in the exterior appearance of aphids imaged with microCT, (Fig 1D, 1E and 1F) but it was clear from the internal structure that the laser treatment effectively sealed the wound with a thick layer of tissue [27].

### Collecting alarm pheromone

**Fig 2:**
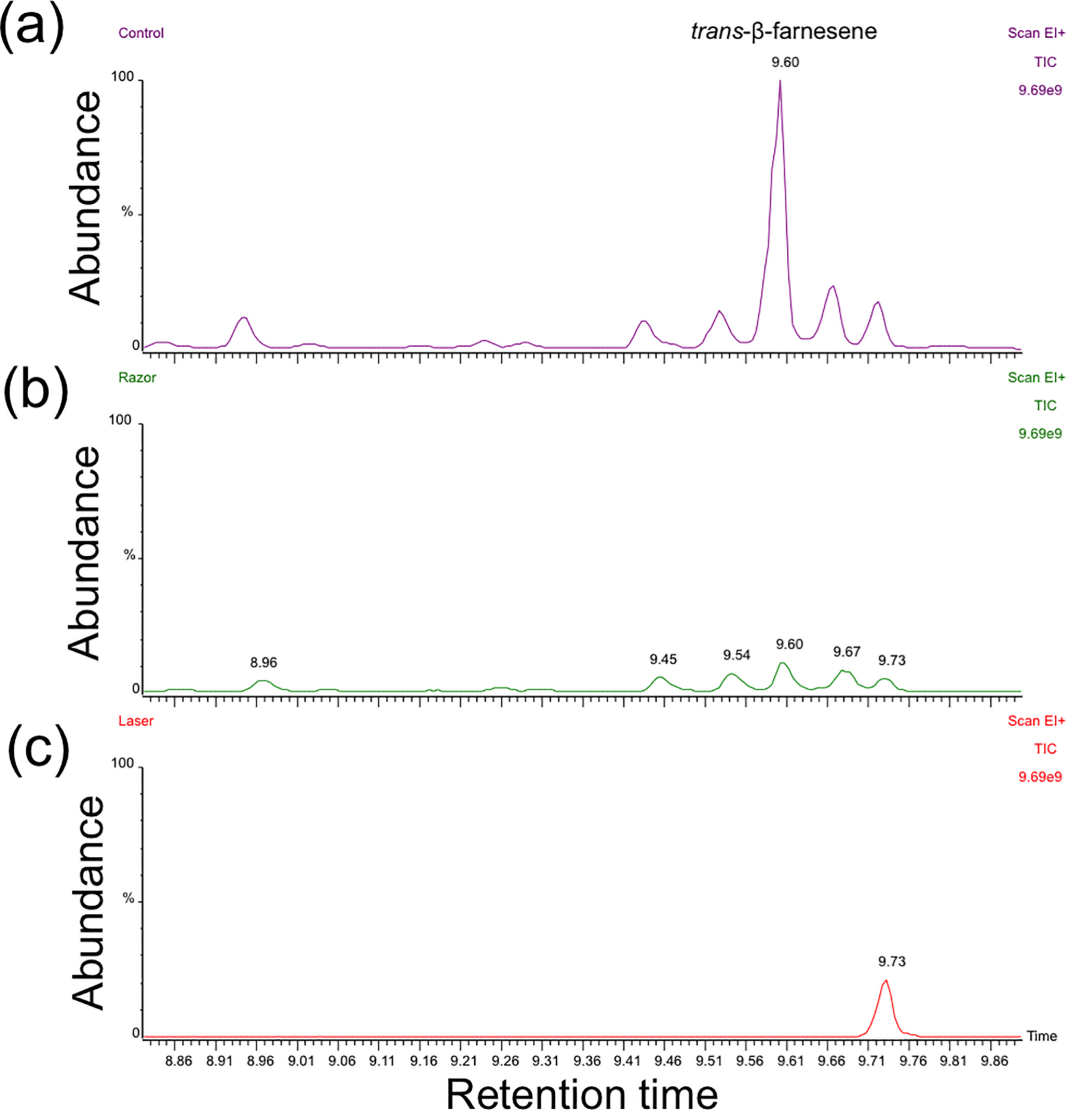
GC-MS analysis of aphid cornicle emissions. GC-MS chromatogram of trans-β-farnesene collected from aphid with intact siphunculi (a). GC-MS chromatogram of trans-β-farnesene collected from razor treated siphunculi (b). GC-MS chromatogram of trans-β-farnesene collected from laser treated siphunculi (cut with razor then sealed with laser beam) (c).

In the control group, trans-β-farnesene was the main volatile identified and it was detected in high quantity at retention time 9.60 min **(**Fig 2A**)**. In the razor group, in which the siphunculi were cut but not sealed, trans-β-farnesene was also detected in much less quantity than in the control treatment **(**Fig 2B**)**. In the laser group, in which the siphunculi were removed and sealed, only a trace amount of trans-β-farnesene was detected **(**Fig 2C).

### Biomechanical inversion experiment

In all treatments, aphids were inverted (i.e. placed on their back) and positioned on the stage of a dissecting microscope. Aphids were observed through the microscope and the total amount of time required to turn back to their upright position (i.e. flipping over) was recorded. In the control treatment, aphids relied completely on their siphunculi to return to their upright position, and they appeared to load all their weight on the two siphunculi to invert. Control aphids took an average of 11±3.3 sec to flip over. In the absence of both siphunculi (razor and laser treatments), aphids could not right itself. In these treatments, aphids relied primarily on their antennae, with minor help of the third pair of legs. Relying on antennae apparently exhausted the aphids, and in nine out of ten replicates, aphids failed to successfully turn over and eventually died. The remaining aphid was able to flip over, although it spent more than 7 min trying to do so. Additionally, this aphid was obviously exhausted and was not able to move or walk normally if it was disturbed further. When only one cornicle was removed, the aphid relied mainly on the remaining cornicle for flipping over with some help of the antennae and the third pair of legs. Seven times out of ten aphids were able to return to their upright position with an average inversion time of 49.7±9.2 sec (S1 Video).

### Fitness experiments

All the data obtained from the survival experiments were analyzed using Kaplan-Meier analysis. Both log rank and Wilcoxon and their P values were calculated and listed below.

#### Fitness of individual aphids

**Fig 3:**
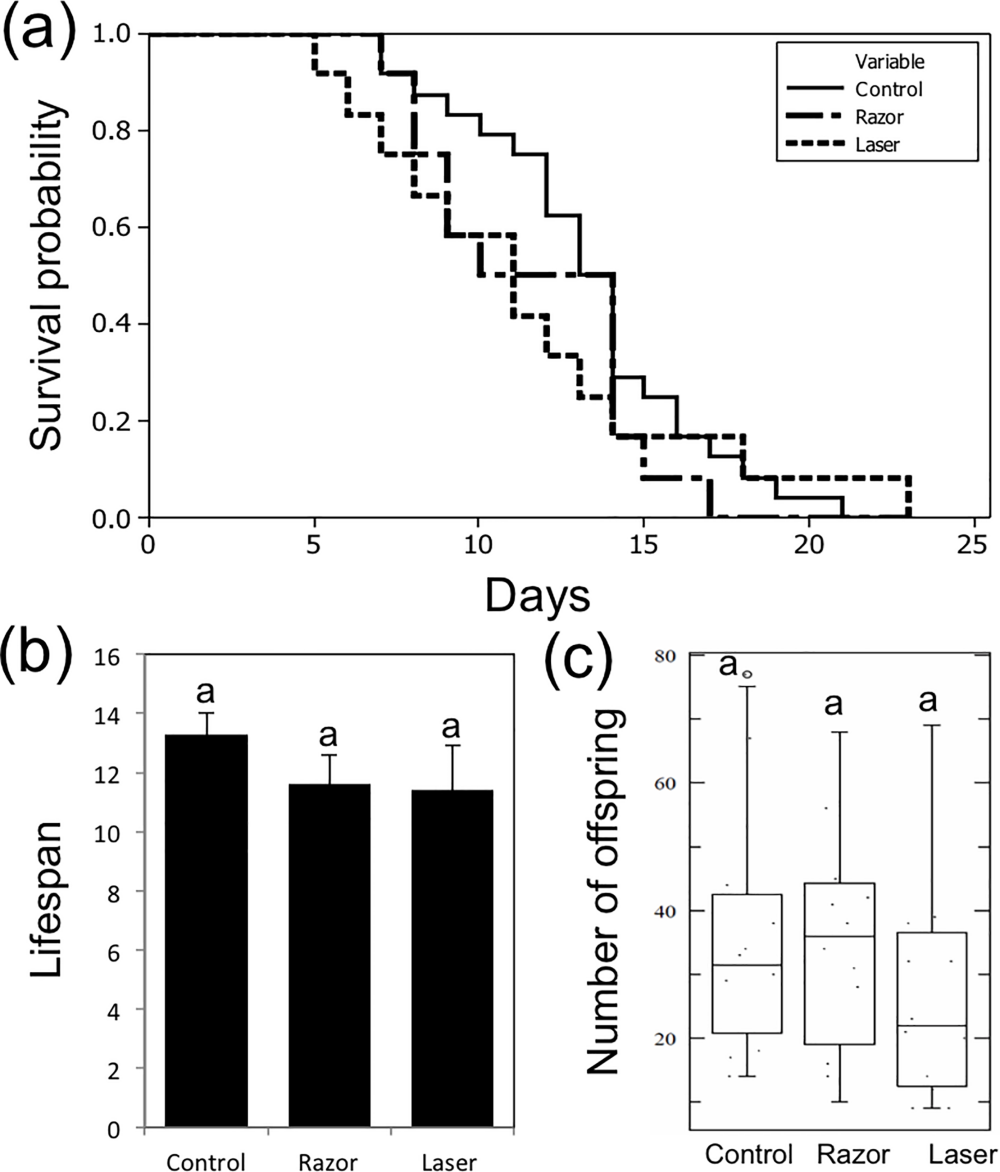
Fitness of an individual aphid. Kaplan-Meier survival curve showing the effect of cornicle removal tools (razor or razor then laser) on lifespan of individual *A*. *citricida* (a). Means of lifespans of *A*. *citricida*. Bars represent the standard deviations; same letters above error bars indicate no significant differences between treatments (*P* > 0.05) (b). Average number of *A*. *citricida* offspring. Boxes show the interquartile ranges including 50% of the values; whiskers reflect the highest and the lowest number of offspring (c). Same letters on box plots indicate no significant differences (*P*> 0.05).

In this experiment, we measured the effects of removing the siphunculi (razor or laser treatments) on the fitness of individual, isolated aphids. Mother survival, number of offspring, aggregation and drop-off behaviors were observed. Kaplan-Meier survival plots showed no significant differences among control, razor, and laser treatments (log rank = 1.0505, *P* = 0.591) (Fig 3A). Aphid lifespan in all treatments was similar with no significant differences among treatments (*P* > 0.05) (Fig 3B). No significant differences in number of offspring were found among all treatments (*P* > 0.05) (Fig 3C). Similarly, we did not observe drop-off in any treatment. These results indicate that cornicle removal by razor and laser did not directly affect the fitness of aphids reared individually in isolation from the colony.

#### Fitness of grouped aphids

**Fig 4:**
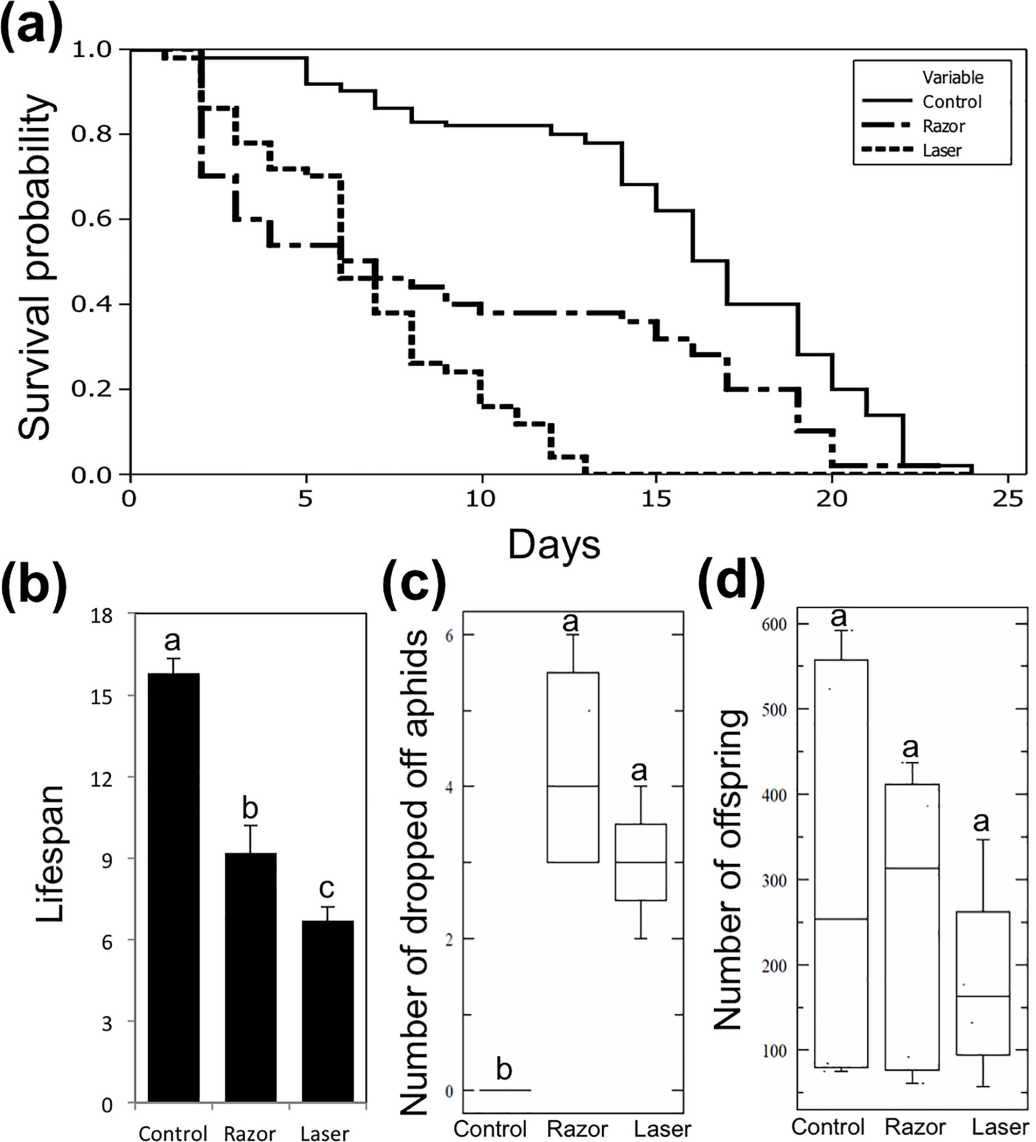
Fitness of grouped aphids. Kaplan-Meier survival curve showing the effect of cornicle removal tools (razor, and razor then laser) on lifespan of grouped *A*. *citricida* (a). Mean of lifespans of *A*. *citricida*. Bars represent the standard deviations; different letters above error bars indicate significant differences between treatments (*P* < 0.05) (b). Average number of dropped *A*. *citricida*. Boxes show the interquartile ranges including 50% of the values; whiskers reflect the highest and the lowest number of dropped *A*. *citricida* aphids (c). Average number of *A*. *citricida* offspring. Boxes show the interquartile ranges including 50% of the values; whiskers reflect the highest and the lowest number of offspring (d). Different letters on box plots indicate significant differences (*P*< 0.05).

In this experiment ten aphids were placed on different leaves of the same plant to investigate the effects of removing the siphunculi on indirect fitness. Mother survival, number of offspring, aggregation and drop-off behaviors were recorded. Kaplan-Meier survival plots showed a significant difference in survival among control, razor, and laser treatments (log rank = 84.8113, *P* < 0.001) (Fig 4A). There was a significant difference in life span among all treatments (*P* < 0.05), with significantly longer lifespan in the control treatment and shortest in the laser treatment, aphids in the razor treatment were intermediate between control and laser-treated aphids (Fig 4B). In drop-off behavior, there was no significant difference between razor and laser groups, while a highly significant difference was found between control aphids and both razor and laser aphids (Fig 4C). Drop-off was low in the control group, while similar numbers of aphids dropped off the plants in both razor and laser treatments.

Furthermore, razor and laser-treated aphids were found to aggregate together on new foliage, while no aggregation behavior occurred in control aphids. Aggregation was found to incur fitness costs to razor and laser-treated aphids. We hypothesize that cornicle removal disrupted chemical communication among treated aphids, and they could not recognize clone-mates and tried to feed on same area. Upon contacting another aphid without siphunculi the aphids dropped off the plants. We did not observe any aphids that successfully dropped off return to the original feeding site, although some were found climbing the plant stem. Number of offspring was similar among all treatments with no significant differences (*P*> 0.05). Therefore, even though fewer adults survived from razor and laser-treated groups, they produced more offspring than aphids with intact siphunculi (Fig 4D). We hypothesize that these aphids increased reproduction in order to save the colony because of the higher mortality and drop-off rate.

#### Fitness of mixed-aphid experiment

**Fig 5:**
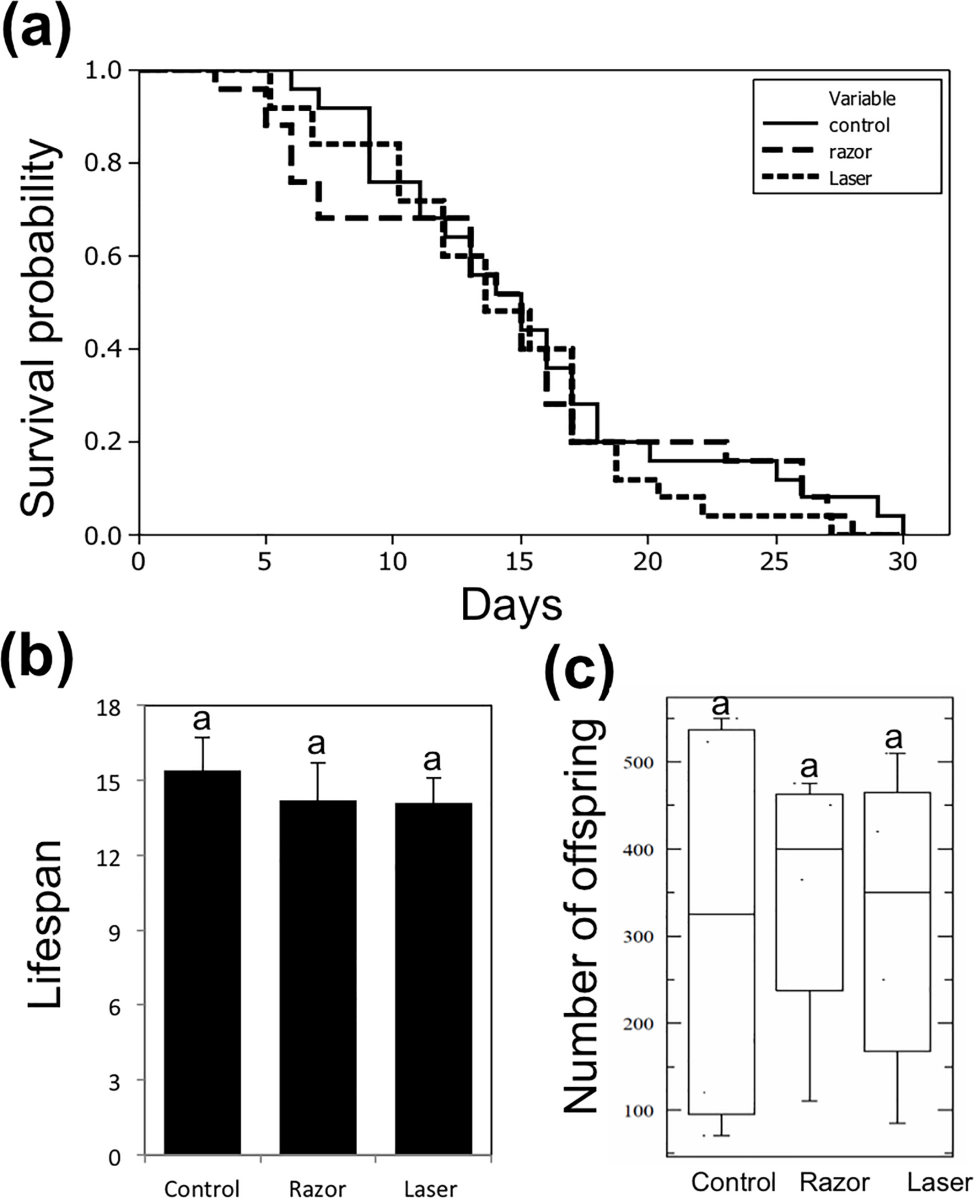
Fitness of mixed aphids. Kaplan-Meier survival curve showing the effect of cornicle removal tools (razor and razor then laser) on lifespans of mixed *A*. *citricida* (a). Average of lifespans of *A*. *citricida*. Bars represent the standard deviations; same letters above error bars indicate no significant differences between treatments (*P* > 0.05) (b). Average number of *A*. *citricida* offspring. Boxes show the interquartile ranges including 50% of the values; whiskers reflect the highest and the lowest number of offspring (c). Same letters on box plots indicate no significant differences (*P* > 0.05).

A group of treated aphids with an equal number of control individuals were placed on the same plants to examine colony communication in the presence of control aphids. In this experiment we investigated mother survival, number of offspring, aggregation and drop-off behaviors. Kaplan-Meier survival analysis indicated no significant differences in survival among control, razor/control, and laser/control treatments (log rank = 0.744, *P* < 0.69) (Fig 5A). These results indicate that the presence of control aphids affected the survival of treated ones positively. No significant differences in lifespan were found among treatments (*P* > 0.05) (Fig 5B). No drop-off behavior was observed in any treatment group. Treated aphids were found to aggregate with control aphids on one leaf. Physical contact did not cause drop-off behavior which suggests that aphids in both razor and laser treatments could recognize their clone-mates in the presence of control aphids, further suggesting that control aphids compensated for the loss of chemical communication among treated aphids. There were no significant differences in the number of offspring in all treatments (Fig 5C).

## Discussion

The evolution and diversification of siphunculi within the Aphididae suggests a strong link between their function and fitness of aphids. Therefore, given the range of reported functions associated with siphunculi, the loss of cornicle function should incur some degree of disruption in communication dynamics and decreases in direct or indirect fitness. Our results show that cornicle removal had no influence on survival or reproduction in isolated aphids, but cornicle removal led to significant fitness costs when aphids were in close proximity and within the effective range of the signaling pheromone trans-β-farnesene emitted from siphunculi. Interestingly, the loss of communication ability in aphids without siphunculi could be recovered by the addition of aphids with fully functional siphunculi to the colony, strongly implicating the trans-β-farnesene in the structuring of the spatial organization of aphids on the host plant. Furthermore, to our knowledge, our data identify siphunculi an important biomechanical structure that they use to flip over after being inverted. So, siphunculi function at both ends of the functional spectrum: they emit the alarm pheromone that initiates drop-off behavior, which may cause them to fall onto their backs, and the siphunculi then serve as a biomechanical fulcrum point for inversion in *A*. *citricida*.

In aphids, chemical signaling occurs through alarm pheromones that are perceived by receptors (sensilla) located on the antennae [2, 16]. Brown citrus aphids of all life stages have been shown to produce the alarm pheromone trans-β-farnesene, but only when they were exposed to an extreme stimulus that threatened the colony [27]. In the current study, trans-β-farnesene was collected in high quantity from control aphids but was also detected in aphids from the razor treatment. We conclude that because the chemical signature of the cornicle emissions was identical in control and razor treatments, but in much lower quantity in the razor treatment, the surgical procedure that removed the cornicle left the area partially obstructed. The SEM and microCT images support this interpretation. On the other hand, we detected almost no trans-β-farnesene emission from aphids with laser-sealed siphunculi, and the complete closure of the opening effectively eliminated the emission of all but one volatile isomer. In both surgical treatments the loss of the cornicle led to reduced signaling and biomechanical capability.

The effects of cornicle removal on individual *A*. *citricida* fitness, when isolated on plants apart from the colony, did not affect aphid survival or number of offspring in both razor and laser treatments. This finding was consistent with results of other studies in which blocking siphunculi did not affect aphid survival [28], or number of offspring [29]. We also found that aphids reared individually did not show escape or drop-off behavior as expected because there was no crowding or interaction with other aphids.

Although cornicle removal did not negatively affect aphid mortality in isolation, the absence of siphunculi was found to cause direct fitness costs to individual aphids in the biomechanical function experiment (i.e. returning to an upright position). In this experiment, the physical function of siphunculi was examined by removing one or both siphunculi completely. Aphids are known to flip on their backs when they run from a feeding area or drop off the plant to escape predation [30]. When an aphid flips over, however, its round, heavy body cannot be supported by its thin legs and antennae. Therefore, the specialized function of siphunculi and cauda appear to be for use as a fulcrum point for flipping over. Utilizing these robust structures is likely more effective than using fragile body parts such as the antennae. Aphids without siphunculi faced difficulty in flipping back to an upright position resulting in death in most cases when both siphunculi were removed. Siphunculi have strong, movable muscles used primarily for directional emission of alarm pheromone [28], but our data suggest that they also enable the aphid to flip over, and highlight the dual-functionality of this structure.

In nature, groups of aphids avoid aggregation in order to reduce the competition for feeding resources. Normally, adult aphids are found feeding solitarily on the most succulent parts of the plant where they will also give birth to their offspring. As the juveniles grow, the older aphids shift to older leaves and stems to make room for the next generation. In high density infestations, or when aphids cover the plant stems and leaves entirely, they communicate with each other chemically, through the use of cornicle secretions and antennae receptors (sensilla). We tested the indirect fitness associated with siphunculi in grouped aphids, by placing ten aphids from each treatment on separate leaves (one aphid per leaf) and then comparing aggregation behavior, lifespan, drop-off rate, and fecundity. In both razor and laser-treated aphids, escape behavior and drop-off rate were increased compared to the control. In both cornicle removal treatments, the surviving aphids were not able to maintain an appropriate distance from other aphids even after placing them on different leaves. We hypothesize that brown citrus aphid without siphunculi became “chemically blind” and were not able to signal their presence among neighboring aphids, resulting in abnormal aggregation behavior. Conversely, in the control treatment, aphids did not aggregate, and each aphid reproduced on different regions of new growth on the plant. Thus, chemical signals emitted and perceived by aphids appear to play a significant role in the demarcation of territory on the plant. Furthermore, the lack of siphunculi adversely impacted indirect fitness because survival was reduced in razor and laser treatments but not in the control treatment, suggesting that physical contact with clone-mates caused by “false” aggregation behavior could not be distinguished from physical attack of natural enemy without the use of the siphunculi. Physical contact between aphids has been shown to cause alarm pheromone signaling [22], however, aphids in this experiment were not able to detect the presence of other aphids in the same feeding area because their signaling capabilities were disabled, and as a consequence did not disperse. Instead, aphids without siphunculi responded to contact with other aphids aggressively, possibly misidentifying them as natural enemies, which resulted in increased escape and/or drop off behaviors, or aggressive behavior such as kicking other aphids off the plant.

Although the survival of grouped aphids without siphunculi was lower than that of control aphids, interestingly, the number of offspring (as a total population) produced by all treatments was similar. This result suggests a higher reproduction rate for cornicle-removed aphids which agrees with a previous study that found aphids produced more offspring during high rates of predation, presumably to enhance colony survival [31]. Therefore, aphids with impaired chemical communications may focus on colony survival by maximizing reproductive investment, highlighting the integral role of chemical communication in aphid colony behavior and their ability to perceive their environment. Aggregation behavior following cornicle removal was found to increase aphid direct fitness as it lowers the chance of being captured by natural enemies [32]. In the current study, no natural enemy was involved, so aggregation behavior was most likely due to loss of chemical signaling among aphids.

In another experiment to test our hypothesis, groups of control and surgically altered aphids were placed on different leaves of the same plant. Two to three surgically altered aphids aggregated with one or two control aphids but moved away after a very short time, and aphids without siphunculi did not drop off the plant. Additionally, there were no significant differences in adult survival between treatments. These results suggest that the effects of cornicle removal could be recovered by the presence of aphids with intact siphunculi and functioning chemical communication, which ultimately led to equivalent survival rates of control aphids and aphids without siphunculi. The production of communication pheromones by control aphids appear to compensate for the loss of chemical communication among surgically altered aphids which survived as long as control aphids were present. Physical contact did not cause drop-off, escape or aggressive behaviors by aphids without siphunculi, suggesting that they were able to differentiate between physical contact of other aphids and that of a natural enemy. Accordingly, the current study suggests that chemical signaling produced by the siphunculi plays an essential role in aphid colony fitness.

Collectively, these data suggest a close linkage between cornicle function and aphid colony communication and biomechanics. The surgical procedures applied here allowed us to control the strength of chemical emissions from siphunculi, and then document the changes in behavior, survivorship and fecundity. Siphunculi clearly play a vital role in the brown citrus aphid, *A*. *citricida* lifestyle by providing physical agility, and chemical communication via alarm pheromones between clone-mates in the aphid colony. Given the diversity of cornicle morphology within the Aphididae, targeted studies on species with varying cornicle properties (e.g. relative length) should allow for further analysis of the relative contributions of siphunculi to overall fitness and their role in biomechanics and chemical signaling.

## Supporting information

S1 Video Biomechanical inversion experiment. In the control treatment, aphids relied completely on their siphunculi (cornicles) to return to their upright position, and they appeared to load all their weight on the two siphunculi to invert. In the absence of both siphunculi (razor and laser treatments), aphids could not right themselves.
(WMV)
